# Necrosis as an Effect of Erysipelas

**Published:** 1865-06

**Authors:** E. Andrews

**Affiliations:** Prof. of Surgery in Chicago Medical College


ARTICLE XXI.

### NECROSIS AS AN EFFECT OF ERYSIPELAS.

By E. ANDREWS, M.D., Prof. of Surgery in Chicago Medical College.

The military amputations presented by returning soldiers display a remarkable number of cases of necrosis of the bones of the stump, and in almost every instance it is found that the patient had erysipelas soon after the amputation. The fact that the poison of erysipelas exerts a very destructive influence upon osseous tissue is a matter of daily observation by surgeons, but the phenomena of the military cases referred to are more curious and interesting than usual.

CASE I. Col. McG——, of an Ohio regiment. He was shot in five places, in a hand-to-hand fight, shattering one arm and fore-arm frightfully. The arm was amputated at the middle, and the operation was followed by erysipelas. The erysipelas having subsided, he waited many months and then presented himself with a fistula in the end of the stump, in which the probe readily discovered a sequestrum. After anæsthetizing the patient, I proceeded to remove the fragment, which I determined to do without re-amputating. On making an incision into the end of the stump, it was found that the whole thickness of the humerus was dead, but that the periostum had produced a new shell of bone around it, and, between the two, a probe easily ran up four inches. The sequestrum was loose, but still held by irregularities of its surface, so that it could not be withdrawn. With a strong scalpel, I laid open longitudinally the soft shell of new bone, cutting it on two sides. With a little force, I then spread these two valves, so as to liberate the sequestrum between them and allow it to be drawn out. The valves were then brought together and dressed, and the cure perfected without shortening the stump in the least. The sequestrum was about four inches in length, and tubular in form. When it was withdrawn, the medullary substance proved to be living, and remained in the wound attached by its superior extremity. The endosteum had produced no shell of bone like the periosteum, but there were numerous osseous spiculæ in the substance of the medulla.

Case II. A soldier of the 1st Ill. Light Artillery, was shot in the arm at Vicksburg, and suffered amputation in the middle of the humerus. After the operation he had erysipelas. Some months later, I examined and found a fistula and sequestrum. I operated in the same manner as in Case I., and found the condition of things exactly a repetition of that case in all particulars. The patient recovered without difficulty.

Case III. Was like Case II., an amputation of the arm followed by erysipelas, The sequestrum, however, was only an inch long, and was removed by a slight operation.

Case IV. Was an amputation above the middle of the arm, followed by erysipelas and necrosis. In this case, the necrosis involved the whole stump of the humerus, quite up to the shoulder-joint. It being presented to me for counsel, I found the patient extremely anxious to preserve the semblance of a stump, even if it was of no practical use. I therefore advised that the bone be removed by resection, saving as much periosteum as possible. This was done, and a good recovery ensued. I do not know to what extent the bone was renewed, as the patient removed to a distance.

Case V. Was shot through the upper-third of the humerus, and had erysipelas soon after. The limb was not amputated. The fracture united well, but caries ensued, involving the shoulder-joint, and accompanied with a bony anchylosis. In this instance I resected the shoulder, after which the patient recovered as usual.

Case VI. Was a gunshot through the ankle, for which the leg was amputated three inches above the ankle. He came to me after some months with a fistula in the end of the stump, and had observed several fragments of bone to pass out. On examination, the bone was found very much thickened and indurated, with a small sequestrum in its very centre, and about six inches above the extremity. Finding that it could not be extracted satisfactorily, I was forced to make a new amputation in the upper-third of the leg. The patient recovered rapidly.

These cases show very clearly that it is seldom necessary to amputate a stump, on account of necrosis of the bone within it. It suffices, generally, to lay open the part and remove the sequestrum, when a good recovery will ensue. They illustrate, also, the destructive effect of the erysipelatous poison upon injured bones, and the consequent folly of hurrying wounded soldiers from the tents of the field of battle, where erysipelas is rarely seen, into large hospitals, where it is a constant attendant, and exerts its fullest and deadliest effect.

